# Designing a synthetic microbial community through genome metabolic modeling to enhance plant–microbe interaction

**DOI:** 10.1186/s40793-023-00536-3

**Published:** 2023-11-16

**Authors:** Osiel S. Gonçalves, Christopher J. Creevey, Mateus F. Santana

**Affiliations:** 1 Grupo de Genômica Eco-evolutiva Microbiana, Laboratório de Genética Molecular de Microrganismos, Departamento de Microbiologia, Instituto de Biotecnologia Aplicada à Agropecuária, Universidade Federal de Viçosa, Viçosa, Minas Gerais Brazil; 2 School of Biological Sciences, Institute for Global Food Security, Queen’s University Belfast, Belfast, BT9 5DL UK

**Keywords:** Microbe-microbe-plant interactions, Plant growth-promoting bacteria, Reverse-ecology, SynComs

## Abstract

**Background:**

Manipulating the rhizosphere microbial community through beneficial microorganism inoculation has gained interest in improving crop productivity and stress resistance. Synthetic microbial communities, known as SynComs, mimic natural microbial compositions while reducing the number of components. However, achieving this goal requires a comprehensive understanding of natural microbial communities and carefully selecting compatible microorganisms with colonization traits, which still pose challenges. In this study, we employed multi-genome metabolic modeling of 270 previously described metagenome-assembled genomes from Campos rupestres to design a synthetic microbial community to improve the yield of important crop plants.

**Results:**

We used a targeted approach to select a minimal community (MinCom) encompassing essential compounds for microbial metabolism and compounds relevant to plant interactions. This resulted in a reduction of the initial community size by approximately 4.5-fold. Notably, the MinCom retained crucial genes associated with essential plant growth-promoting traits, such as iron acquisition, exopolysaccharide production, potassium solubilization, nitrogen fixation, GABA production, and IAA-related tryptophan metabolism. Furthermore, our in-silico selection for the SymComs, based on a comprehensive understanding of microbe-microbe-plant interactions, yielded a set of six hub species that displayed notable taxonomic novelty, including members of the Eremiobacterota and Verrucomicrobiota phyla.

**Conclusion:**

Overall, the study contributes to the growing body of research on synthetic microbial communities and their potential to enhance agricultural practices. The insights gained from our in-silico approach and the selection of hub species pave the way for further investigations into the development of tailored microbial communities that can optimize crop productivity and improve stress resilience in agricultural systems.

**Supplementary Information:**

The online version contains supplementary material available at 10.1186/s40793-023-00536-3.

## Background

Plants and microbial communities have a complex and interdependent relationship, involving various mechanisms that influence ecological interactions. One crucial aspect is the release of photosynthates by plants belowground through mucilage and exudates that serve as energy sources for specific microbial groups [1–3]. In return, certain microbial taxa can positively impact plant growth and protect against biotic and abiotic stresses [4]. They achieve this through the synthesis of phytohormones, nutrient acquisition, and by engaging in antagonistic interactions with plant pathogens [5, 6]. Consequently, it is intriguing to consider that plants experiencing drought can dynamically influence the eco-evolutionary dynamics of the microbial community and thereby affect plant performance [7].

In recent years, there has been growing interest in harnessing the potential of microbial communities in the rhizosphere to improve crop productivity and enhance stress resistance [8–10]. One approach gaining attention is the manipulation of the rhizosphere microbial community through the inoculation of beneficial microorganisms. This strategy aims to establish synthetic microbial communities (SynCom) that can positively influence plant health and yield. SynCom aims to mimic the original microbial composition by reducing the number of components in the community while preserving the essential characteristics of their natural counterparts [11].

The designing of SynCom that promotes plant growth and helps plants withstand various stresses, such as drought, salinity, and pathogen attacks, relies upon a careful selection and compatibility of specific microorganisms into the community that possesses traits for robust colonization, prevalence throughout plant development and specific beneficial functions for plants [12–16]. Genome-scale metabolic networks (GSMNs) play a pivotal role in elucidating the intricate metabolic interactions between microorganisms and their host plants [17–19]. GSMNs serve as comprehensive computational models, encapsulating the entirety of metabolic reactions and pathways present in microbial genomes [20]. Therefore, by reconstructing GSMNs for individual microorganisms and assessing their collective metabolic potential, we gain invaluable insights into how these microorganisms contribute to the overall ecosystem function.

Decades of extensive cultivation-independent study, as well as recent high-throughput sequencing technology, have radically altered our perspectives on the diversity of microbial life [21, 22]. These approaches capture the breadth of bacterial and archaeal genomic diversity across Earth’s biomes and provide a resource that underscores the value of genome-centric approaches for revealing genomic properties of uncultivated microorganisms that affect ecosystem processes [23]. However, there are still significant limitations in answering the fundamental ecological and evolutionary questions surrounding natural microbial communities. In addition, genome-resolved metagenomics represents a valuable strategy for acquiring insights into the genomic properties, metabolic capabilities, and functional potential of individual microorganisms within complex microbial communities [24–27]. This enables a deeper understanding of their ecological roles, interactions, and contributions to various environmental processes.

The Cerrado, known for its arid conditions and nutrient-poor soils, harbors a diverse array of resilient plants with an associated microbiome [28–30]. In addition, the Campos rupestres, a distinctive and biodiverse rocky outcrop ecosystem found in the Cerrado, contribute to the unique microbial communities and plant adaptations in this region. Therefore, harnessing novel soil bacteria from harsh environments like the Brazilian Cerrado holds immense potential for agricultural practices. These novel plant-promoting bacteria can enhance the growth, productivity, and stress tolerance of important crop plants such as soybean, maize, sorghum, and sugarcane. Notably, their ability to mitigate drought stress in these crops is of utmost importance, considering the increasing frequency of drought events caused by climate change [31, 32].

In this study, our aims were threefold: (1) to reconstruct GSMNs to gain insight into the metabolic complementarity between bacterial species and host crop plants; (2) to define a minimal microbiota community; and (3) to select hub-species that preserve the essential plant growth-promoting traits (PGPTs). To this end, we integrated several in silico approaches, employing multi-genome metabolic modeling of 270 previously described metagenome-assembled genomes (MAGs) from Campos rupestres, a grassland ecosystem located in Brazil.

## Materials and methods

### Genome data

A total of 270 MAGs were retrieved, belonging to two dominant plant species *Vellozia epidendroide*s and *Barbacenia macrantha* found in the Campos rupestres [33]. Annotated MAGs were downloaded from the National Center for Biotechnology Information (NCBI) under the BioProject PRJNA522264. MAGs were filtered based on co-assembly type to prevent data redundancy. MAG details are provided in Additional file 2: Table S1.

We applied CheckM v1.0.13 [34] with the tree_qa command to extract a set of 43 single-copy, protein-coding marker genes. These marker genes were employed to evaluate phylogenetic markers within a dataset consisting of 270 assembled metagenomic bins. The concatenated protein alignments of the 43 universal marker genes, obtained from CheckM, were then used to reconstruct a maximum-likelihood phylogenetic tree using IQ-TREE v1.6.11 [35]. During the reconstruction process, specific parameters ('*-m TEST -bb 1000'*) were implemented to ensure the generation of an accurate tree. Subsequently, the phylogenetic tree was uploaded to iTOL [36], where it underwent visual annotation, including color coding and the application of heatmaps.

### Reconstruction of the genome-scale metabolic networks (GSMNs)

The metabolic networks of each MAG were analyzed using genome-scale metabolic models reconstructed through an automated command-line version of PathwayTools [37]. The entire analysis was conducted using the metage2metabo (m2m) tool suite [38]. To begin the reconstruction process, we utilized the *mpwt* (multiprocessing pathway tools) program from the m2m tool with the "–path" flag to create a PathoLogic environment for each genome in gbff format. Subsequently, we performed the automatic reconstruction of non-curated metabolic networks using the *m2m recon* command line.

For the analysis of metabolic producibility and calculation of cooperation potential, the *m2m iscope* command line was employed for individual potential, and the *m2m cscope* command line for collective metabolic potentials. Additionally, in the *m2m cscope* command, the "-m" flag was added to incorporate the host metabolic network in the SBML file format. The SBML files for maize (*Zea mays*), sugarcane (*Saccharum officinarum*), and sorghum (*Sorghum bicolor*) were obtained from C4GEM [39]. Furthermore, for soybean (*Glycine max*), non-curated GSMNs were reconstructed using the *m2m recon* command line for the genome of the *G. max* cultivar EMBRAPA BRS 537, downloaded from NCBI (Supplementary data 1). These plants were selected based on their significance as major crops with economic importance and global relevance. These crops are staples in many regions and contribute significantly to food and bioenergy production.

As a nutritional constraint, root exudate-mimicking growth media was used [19, 40], which were implemented as a "seed" (-s flag in m2m) for the targeted predicted producible metabolites (Additional file 2: Table S2).

The selection of the minimal community and computation of key species were performed using the *m2m mincom* command line. For this analysis, each host GSMN in SBML format was considered, including essential compounds for metabolism such as amino acids, nucleotide components, cofactors, vitamins, phytohormones, organic acids, and other compounds relevant to plant interactions (Additional file 2: Table S3). The identification of key species for the targeted set of compounds was carried out using MiSCoTo [41], which is implemented in the m2m suite.

The MetaCyc Metabolic Pathway Database [42] and the Kyoto Encyclopedia of Genes and Genomes were used as references to link genome annotation to metabolism. The data visualization was accomplished using the *ggplot2* package in the R programming environment.

### Identification of plant growth-promoting traits (PGPTs)

To identify genes linked to plant growth-promoting traits (PGPTs) within the MAGs, an alignment of their protein sequences was performed. This alignment was performed using a combination of BLASTP and HMMER tools available in the PGPT-Pred database from PLaBAse [43]. To ensure the accuracy of the annotations, we further validated them by conducting BLASTP searches against the NCBI non-redundant protein, RefSeq, and UniProtKB/Swiss-Prot databases. Hits with an E-value < 1e−5 were considered significant for both approaches.

To evaluate the potential interaction of the MAGs with plants, a criterion was established based on the presence or absence of 86 PGPT genes. These genes are involved in nitrogen fixation, phosphorus solubilization, as well as the production of exopolysaccharide (EPS), siderophores, and plant growth hormones. Specifically:

- Nitrogen-fixing genes: *nifA, nifB, nifD, nifE, nifF, nifH, nifHD1, nifHD2, nifJ, nifK, nifM, nifN, nifQ, nifS, nifT, nifU, nifV, nifW, nifX, nifZ.*
- Exopolysaccharide (EPS) production*: epsE, epsD, epsF, epsH, epsI, epsJ, epsL, epsM, epsN, epsO.*
- Root colonization by nodulation: *nodA, nodB, nodC, node, nodF, nodI, nodJ, nodU, nod, nodT, nolM, noeA, noeB, noeC, noeD, noeE, nodN, nodN_like, nodO, nodP, nodS, nodS_like, nodY, nodZ, nodV, nodV_like, nodW, nodX, nodX, nodO.*
- Oxidative stress|ROS scavenging: *sodN, sodC, sod3.*
- Iron acquisition: *lipA, lipB, lipL, lipL2, lipM, lplA.*
- Salinity stress-potassium transport: *kdpA, kdpB, kdpC, kdpD, kdpE, kdpF.*
- Plant embryogenesis-spermidine: *puuA, puuB, puuC, puuD, puuE, pup*
- IAA-related tryptophan metabolism*: trpA, trpB, trpC, trpCF, trpD, trpE, trpEG, trpG, trpDG, trpF, trpS, trpR*

The data visualization was accomplished using the *ggplot2* package in the R programming environment.

### Microbe–microbe and plant–microbe interactions using reverse ecology

Protein-level assemblies of eight MAGs were annotated using KofamKOALA [44] (KOfam parameters: '–e-value 0.00001'). The KEGG orthologs (KO) obtained from these annotations were utilized for determining microbe-microbe interactions, specifically competition and complementarity indices. To calculate these indices, the Cooperation Index package in RevEcoR was employed [45]. Candidates with criteria scores exceeding 0.6 were considered potential competitors.

Next, a matrix that included the substrate and product information for each species from the RevEcoR analysis was created. We further applied NetCooperate to gain insights into potential ecological host-microbe interactions [46]. For pairwise interactions between hosts and microbes, the biosynthetic support score (BSS) and the metabolic complementarity index (MCI) were measured. The KOs obtained from the annotated host genomes, which were sourced from the Joint Genome Institute (JGI, https://genome.jgi.doe.gov/portal/), were considered for these analyses. The compounds involved were annotated in KEGG compounds with their biological roles and the Phytochemical Compounds Database (https://www.genome.jp/kegg/ compound/) as well as the MetaCyc Metabolic Pathway Database.

## Results

### Design of this study

In this study, we investigated 270 previously described MAGs obtained from Campos rupestres, a grassland ecosystem located on the geologically ancient rocky outcrops of central and eastern regions of Brazil [33]. This unique ecosystem is characterized by extremely low concentrations of essential nutrients, creating challenging conditions for plant growth [47]. However, it also serves as a habitat for microorganisms that have adapted to these harsh conditions. The MAGs were primarily sourced from bulk soil (BS) and the rhizosphere (RX) of two dominant plant species in the Campos rupestres, namely *V. epidendroide*s (BE) and *B. macrantha* (BM).

**Fig. 1:**
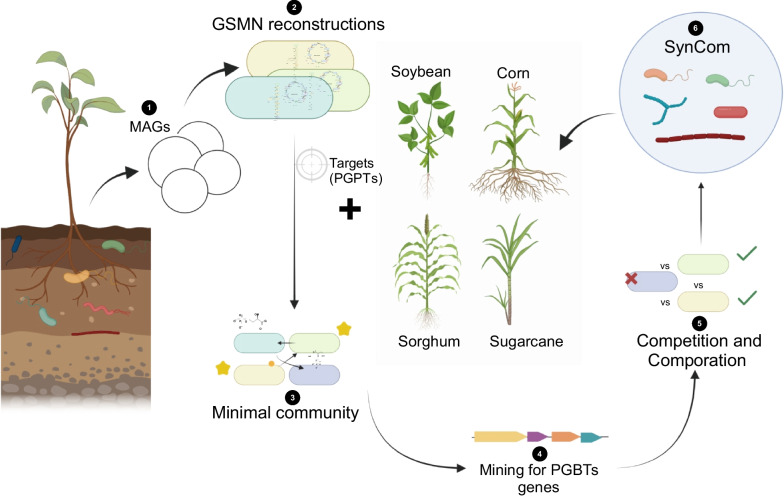
The workflow employed in this study to design the SymCom. (**1**) The study starts with the use of 270 MAGs obtained from the microbiomes associated with *V. epidendroides* and *B. macrantha* in Campos rupestres, as detailed in Camargo et al. [47]. (**2**) Subsequently, genome-scale metabolic networks (GSMNs) of individual metagenome-assembled genomes were reconstructed using the M2M tool suite. To determine the target compound, which encompasses crucial compounds for their metabolism (such as amino acids, nucleotide components, cofactors, vitamins, phytohormones, organic acids, and other compounds relevant to plant interactions), as well as by incorporating GSMNs of significant crop plants, (**3**) we curated a minimal community (MinCom) from the original microbiome community linked to *V. epidendroides* and *B. macrantha*. From this MinCom, we identified genes associated with plant growth-promoting traits (PGPTs) (**4**) and employed a reverse ecology approach (**5**) to select species that collectively constitute our SymCom **(6)**. The figure was designed using BioRender

To gain insights into the metabolic capabilities of these microorganisms, GSMNs were reconstructed for the MAGs and determined the minimum number of species required to perform specific metabolic functions related to plant–microbe interactions. We considered a predefined set of target compounds and incorporated information about the associated plant hosts. By doing so, the complexity of the microbiota was successfully reduced, and minimal communities with comparable properties were identified (Fig. 1).

Furthermore, a comprehensive analysis of key species within these minimal communities was conducted, with a focus on their PGPTs. Additionally, competition and complementarity indices were calculated for each pair of species to assess their potential interactions. This thorough workflow allowed us to identify beneficial microbes and construct SynCom that have the potential to enhance crop productivity (Fig. 1).

### Soil and rhizosphere microbiome associated with dominant plant species in the Campos rupestres

**Fig. 2:**
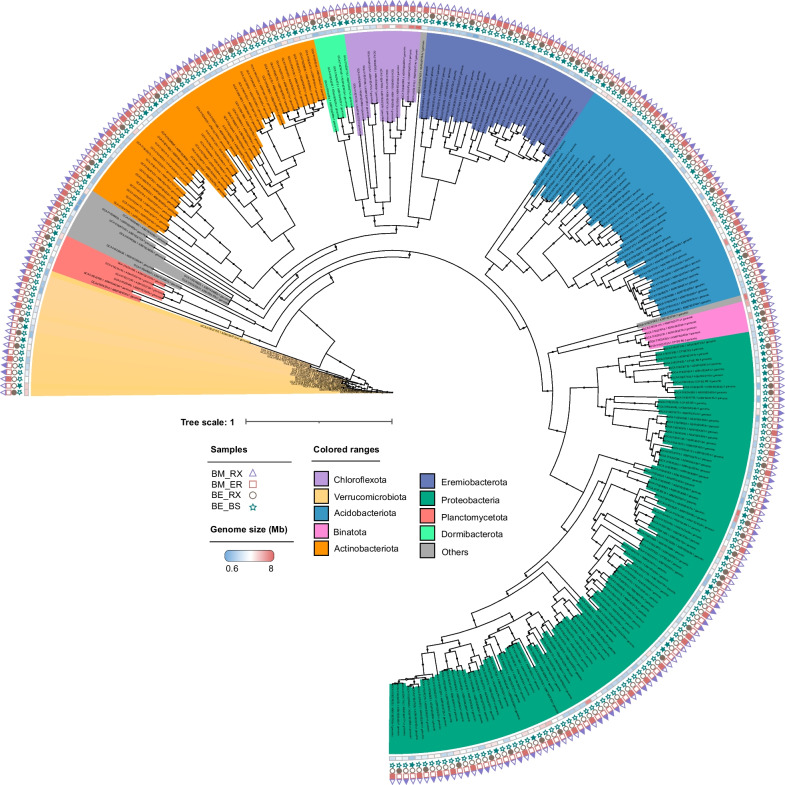
Phylogeny of 270 metagenome-assembled genomes (MAGs) from Campos rupestres. A maximum likelihood phylogenetic tree based on 43 single-copy; protein-coding marker genes identified using CheckM. Phyla are marked in different background colors. The first arc of the tree represents the genome size of each MAG, followed by sequential arcs indicating the source of the obtained MAGs, distinguished by filled geometric forms. A phylogenetic tree was built under the model of rate heterogeneity G + F + I + G4, and a maximum of 1000 bootstrap replicates. Bootstraps are shown in black circles. The tree is drawn to scale, with branch lengths in the same units as those of the evolutionary distances used to infer the phylogenetic tree. Colored-filled geometric forms in the samples represent the presence of microbial taxa in those samples. Abbreviations: BE_BS (bulk soil of *V. epidendroides*), BE_RX (Rhizosphere of *V. epidendroides*), BM_BS (bulk soil of *B. macrantha*), and BM_RX (rhizosphere of *B. macrantha*)

Here, 270 MAGs were obtained from the soil and rhizosphere microbiomes of *V. epidendroides* (BE) and *B. macrantha* (BM). Among the MAGs, those belonging to the phylum Proteobacteria were the most abundant in the samples (37.4%), followed by Actinobacteriota (15.9%) and Acidobacteriota (14.8%) (Fig. 2, Additional file 1: Fig. S1A). Interestingly, a significant presence of the phylum Eremiobacterota was observed. This phylum is known for its ecological versatility and ability to thrive under various extreme environmental conditions [48], which accounted for 10.3% of the species in the dataset used in this study.

The size of the MAGs varied, ranging from 0.6 Mb in a MAG affiliated with the phylum Patescibacteria to 8 Mb in MAGs belonging to Chloroflexota, Proteobacteria, and Myxococcota (Fig. 2, Additional file 1: Fig. S1B). Generally, there was no observed direct relationship between the distribution of phyla and the environment. Most MAGs were widely distributed throughout the Campos rupestres, which may be attributed to the heterogeneity of MAGs within and across phyla. A total of 48 MAGs were obtained from the bulk soil of *V. epidendroides* (BE_BS), 55 MAGs from the rhizosphere of *V. epidendroides* (BE_RX), 82 MAGs from the bulk soil of *B. macrantha* (BM_BS) and 85 MAGs from the rhizosphere of *B. macrantha* (BM_RX). For detailed information on each MAG and its taxonomic assessment, please refer to Additional file 2: Table S1.

### Reconstruction of genome-scale metabolic networks (GSMNs) to understand metabolic complementarity between species

Pathway Tools [37] integrated within the m2m [38] were used to identify metabolic functions and species of interest within the Campos rupestres microbiota. Notably, the overall statistics of GSMNs varied depending on the number of MAGs per sample, with MAGs from BE_BS displaying relatively lower numbers. In total, the reconstruction of GSMNs encompassed a range of 59,479 to 101,411 compounds, 39,700 to 72,566 reactions, and 945 to 1742 pathways. MAGs from different samples exhibited a similar number of pathways (Additional file 1: Fig. S2, Additional file 2: Table S4, Table S5). Individual GSMNs for each MAGs are provided in Suplementary Data 1.

**Fig. 3:**
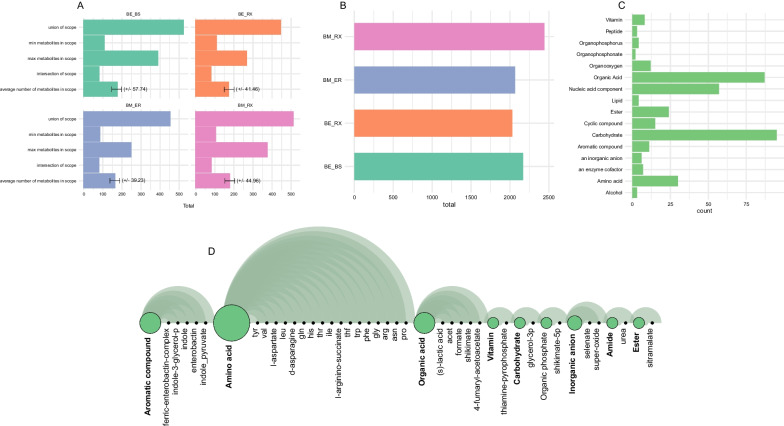
Metabolic producibility and cooperation potential of the microbiome from Campos rupestres. **A** Metrics displaying individual metabolic potentials (scopes), showcasing the minimum, maximum, and arithmetic mean number of compounds across all scopes for each sample collection. Sample IDs are indicated above each graph bar. **B** The total number of producible compounds accessible to the community in each sample. **C** Classification of the accessible compounds into categories within the community. **D** Detailed information regarding the target compounds accessible to the entire community. Circles represent the proportion of each class, while arcs depict the set of compounds within each class. *BE_BS* bulk soil of *V. epidendroides*, *BE_RX* Rhizosphere of *V. epidendroides*, *BM_BS* bulk soil of *B. macrantha*, *BM_RX* rhizosphere of *B. macrantha*

Next, insights into the metabolic potential, referred to as scopes, of individual metabolic networks, were acquired under specific nutritional conditions, specifically a seed representing a "root exudate-mimicking growth media" (see “Materials and Methods” section for details). Overall, the sizes of individual scopes remained relatively stable across all GSMNs. The results revealed that, on average, each microbiota from the samples could access approximately 80 metabolites (as indicated by the intersection of scopes) (Fig. 3A, Additional file 2: Table S6). When considering the reachable metabolites for all organisms together, the combined scope comprised 521, 444, 451, and 510 metabolites in the BE_BS, BE_RX, BM_BS, and BM_RX samples, respectively (Fig. 3A, Additional file 2: Table S6).

Furthermore, a metabolic potential analysis was carried out, considering the metabolites accessible to the entire community, excluding the seeds. In total, the composition of 519 newly producible metabolites accessible to the community was examined (Fig. 3B, Additional file 2: Table S7). Interestingly, the number of these metabolites remained consistent regardless of the inclusion of the GSMNs of the host plant in the analysis (Additional file 1: Fig. S3) (see “Materials and methods” section for details). Most of these metabolites predominantly consisted of carbohydrates, organic acids, nucleotide components, and amino acids (Fig. 3C). Among the target compounds included in the analysis of the entire community, we found that this community could produce 17 amino acids, five organic acids, and five aromatic compounds (Fig. 3D, Additional file 2: Table S8).

### Defining a minimal community of microbial

The microbiota was reduced into minimal communities with equivalent properties, aligning with the rationale that these communities mimic carefully selected microbial species to fulfill specific microbiome functions. Plant-associated microbial communities adhere to defined phylogenetic organization and general community assembly rules. This approach enables the controlled and targeted analysis of plant–microbe interactions, providing insights into key players and their roles within these ecosystems [49].

**Fig. 4:**
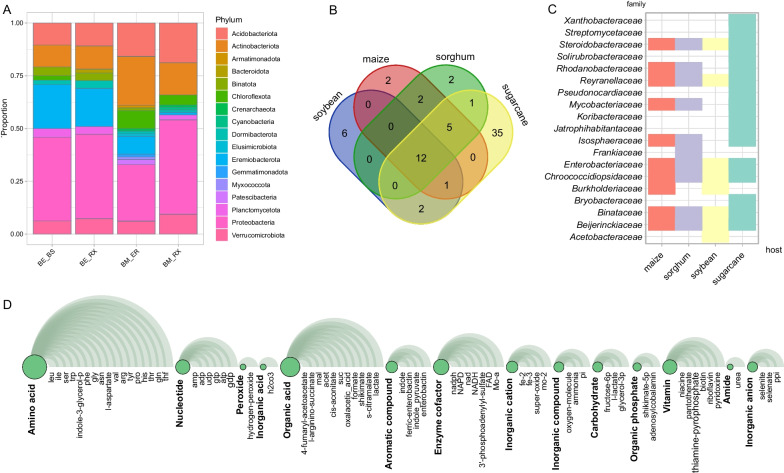
Metabolic producibility of the minimal community of the microbiome from Campos rupestres. **A** Taxonomic abundance of the minimal communities for each sample collection. *BE_BS* bulk soil of *V. epidendroides*, *BE_RX* Rhizosphere of *V. epidendroides*, *BM_BS* bulk soil of *B. macrantha*, *BM_RX* rhizosphere of *B. macrantha*. **B** The core set of species shared among different plant hosts of the reduced community. **C** Distribution of taxonomic family across different plant hosts. **D** Community reduction analysis of the target categories in microbiome from Campos rupestres

Taking into account our desired target compounds and the host metabolism (crop), the community was narrowed down to 68 bacterial species. To be specific, 16 species were associated with soybean, 18 with maize, 17 with sorghum, and 39 with sugarcane (Additional file 2: Table S9). Among these species, 47 MAGs belonged to Proteobacteria, 13 MAGs belonged to Actinobacteriota, 7 MAGs belonged to Eremiobacterota, and 4 MAGs belonged to Acidobacteriota. These phyla were generally present across all samples, but Eremiobacterota showed a stronger association with *V. epidendroide*s (BS/BS), while Actinobacteriota and Acidobacteriota exhibited a preference for *B. macrantha* (Fig. 4A). We also observed a core set of species shared among different plant hosts, with sugarcane hosting a unique set of species (Fig. 4B). At a lower taxonomic level, families such as *Beijerinckiaceae*, *Binataceae*, *Chroococcidiopsidaceae*, *Enterobacteriaceae*, *Reyanellaceae*, and *Steroidobacteraceae* were found to be widespread across all hosts. Sorghum had the highest number of unique families selected (Fig. 4C). Additionally, the producible set of metabolites from this minimal community included essential amino acids, a selection of ten organic acids, aromatic compounds like indole, vitamins, and inorganic ions (Fig. 4D, Additional file 2: Table S10).

**Fig. 5:**
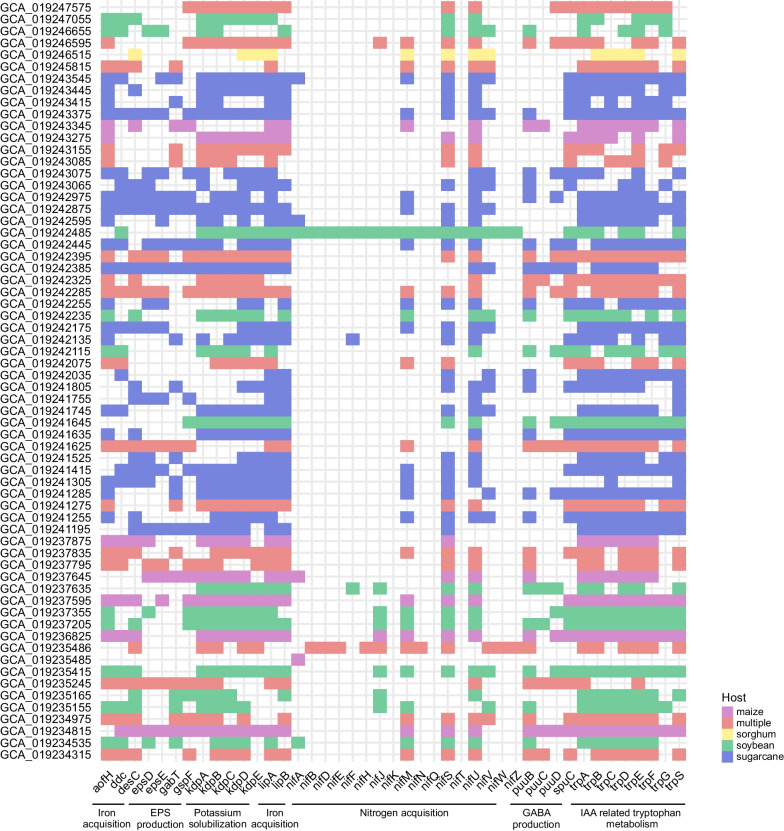
The distribution of key genes associated with plant growth-promoting traits among members of the minimal community in the microbiome from Campos rupestres. The Y-axes represent the GenBank access numbers of individual members within the reduced community, while the X-axes group the PGPT genes into categories based on their shared functions. The graph is color-coded according to the host plant, and the absence of color in squares indicates the absence of the gene. The presence of multiple, red-colored squares indicates genes found across more than two hosts. Details of all PGPT genes are in Additional file 2: Table S11 and Supplementary Data 2

We analyzed the MAGs to identify genes encoding proteins related to plant growth-promoting traits (PGPTs). These PGPT genes were categorized into seven classes, including Iron acquisition, EPS production, potassium solubilization, nitrogen fixation, GABA production, and IAA (indole-3-acetic acid) related tryptophan metabolism (Fig. 5). It is noteworthy that most of these MAGs contained genes encoding PGPT proteins, although the presence and abundance varied across different host plants. Genes encoding for potassium solubilization, and IAA metabolism were more abundant across the MAGs. Specifically, only a few MAGs from the species *Metakosakonia intestini* exhibited the potential for nitrogen fixation. Furthermore, we identified one MAG associated with soybean that possessed a complete set of nitrogen-fixing genes (Fig. 5). Detail of PGPTs for each MAGs can be found in Additional file 2: Table S11 and Supplementary Data 2.

### Designing a SymCom based on key species in a minimal community through pairwise interactions

Within the 68 species of the minimal community, eight species were identified as essential symbionts. These species were consistently present in all minimal communities, thereby facilitating the producibility of the target metabolites [38]. These essential symbionts represented four phyla (Cyanobacteria, Eremiobacterota, Proteobacteria, and Verrucomicrobiota), with only one MAG assigned at the species level, *M. intestine*. Interestingly, these essential symbionts were associated with all crop hosts (Additional file 2: Table S12).

**Fig. 6:**
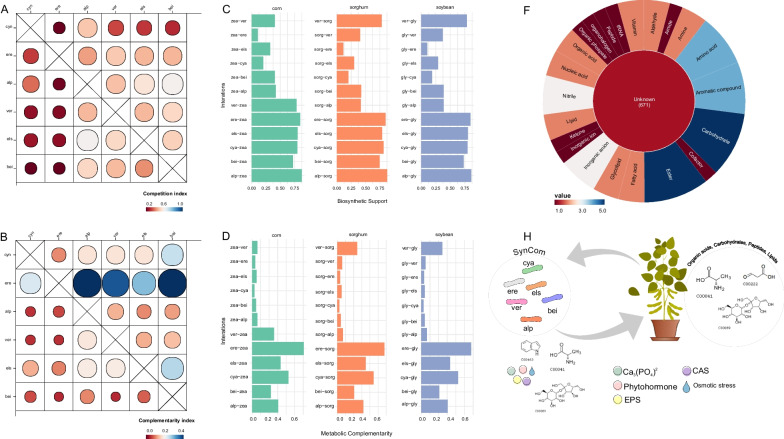
A SymCom designed based on comprehensive knowledge of microbe-microbe-plant interactions. **A** Matrix plot of the competition index among six species of SymCom. The competition index ranges from 0 (indicating the lowest competition) to 1 (representing the highest competition index). **B** Matrix plot of the complementary index among six species of SymCom. The size and color of each circle correspond to the competition and complementary index. **C** The distribution of the biosynthetic support score for the hosts and microbial species. **D** The distribution of the metabolic complementary score for the hosts and microbial species. The Y-axes represent the pairwise interactions and indicate that the first microbe/plant is metabolically supported or complemented by the second microbe/plant. **F** Biosynthetic support with biological pathways found in the compounds of the bacteria-host interaction. The color of each circle corresponds to its abundance, except for the inner circle, which shows the number of each compound for that category. **G** The SymCom was designed with six microbial species showing potential PGPT features that can enhance plant growth. In turn, the host plant may supply potential compounds to the bacteria to support this interaction. *alp* (Alphaproteobacteria), *cya* (Cyanobacteria), *bei (* *Beijerinckiaceae)*, *els* (Elsterales), *ere* (Eremiobacterota), *ver* (Verrucomicrobiota)

We aimed to construct a microbial consortium by identifying microbe-microbe interactions. To assess the dynamics within the consortium, the competition and complementarity indices for all pairs of species were calculated using RevEcoR [45] (Additional file 1: Fig. S4). Among the species in the SymCom, members of Enterobacteriaceae, specifically *M. intestine* and *Enterobacter* sp., exhibited the highest competitiveness index compared to other species. Consequently, we excluded these two species and recalculated the competition index. Interestingly, the results revealed that none of the species exceeded a competition index greater than 0.6, indicating a lack of competition among the species (Fig. 6A).

Regarding the complementarity index, a member of the phylum Eremiobacterota tended to provide support to most members in the SymCom (Fig. 6B). Furthermore, we assessed the metabolic complementarity between species and the host plants (Fig. 6C, D). Initially, the Biosynthetic Support Score (BSS) was computed to assess the host species' capability to fulfill the nutritional needs of a parasitic or commensal species. Furthermore, the Metabolic Complementarity Index (MCI) was calculated to quantify the extent to which two microbial species can support each other through biosynthetic complementarity [17].

The distribution of BSS values across the hosts ranged from approximately 0.1 to 0.8, while the MCI values ranged from 0.0 to 0.4 (Fig. 6A). As expected, there were clear differences in BSS and MCI scores between bacteria and hosts, indicating that hosts tend to better fulfill the nutritional needs of the soybean. Interestingly, a member of the Chthoniobacterales order, belonging to the Verrucomicrobiota phylum, exhibited high BSS and MCI scores in pairwise interactions with sorghum and soybean (Fig. 6C, D). A total of 641 BSS compounds were supported by SymCom and 1,037 compounds in which SymCom was supported by the hosts (Additional file 2: Table S13). Most of these compounds were not assigned to known compounds, but we found that carbohydrates, esters, amino acids, and aromatic compounds were the most frequently involved in the interaction between the SymCom and the hosts (Fig. 6F). Conversely, amino acids, lipids, organic acids, and coenzymes were found to be involved in the interaction between hosts and the SymCom (Additional file 1: Fig. S5).

We have confirmed that the six species included in the SymCom encoding PGPT proteins are actively involved in various essential processes such as nitrogen fixation, phosphorus solubilization, EPS production, siderophore production, and plant growth hormone production (Additional file 2: Table S14) Our observations indicate that the *Beijerinckiaceae* (bei) species possess a comprehensive set of genes responsible for phosphate transport, homeostasis, and degradation (*pho*, *pts*, and *phn* clusters), as well as the production of siderophores like enterobacterin and mycobactin (*ent* and *mdt* clusters). Furthermore, all the species exhibit the potential to promote plant germination through the production of H_2_S and the synthesis of IAA. They also possess the ability to solubilize potassium, with three of them capable of producing the GABA phytohormone and protecting against osmotic stress through the production of osmolytes such as glycine and betaine (Additional file 2: Table S14). Collectively, our findings provide strong evidence that the SymCom model can generate crucial PGPTs that significantly enhance crop productivity. In return, these enhanced plants can contribute to the maintenance and sustainability of SymCom (Fig. 6H).

## Discussion

The integration of omics data acquisition and analysis, along with modeling approaches, enables computational predictions of an organism's resource utilization, biosynthetic capacities, limitations, and growth under diverse conditions [50, 51]. These models rely on the reconstruction of metabolic networks from annotated genomes, which integrate all the expected metabolic reactions of an organism. This makes it possible to predict fundamental information regarding the competition of members of the microbial community, and their cooperation among microbes and their host [52, 53]. Despite the limitations of draft genome-scale metabolic reconstructions [54, 55], they represent valuable starting points for understanding an organism's metabolic potential, but we recognize refinement and validation are necessary to overcome these limitations.

In this study, we used 270 MAGs derived from the microbiomes associated with *V. epidendroides* and *B. macrantha* in campos rupestres, as described by [33]. This unique ecosystem is characterized by remarkably low concentrations of essential nutrients, creating challenging conditions for plant growth. However, these conditions also present an excellent opportunity to explore plant–microbe interactions in harsh environments. The authors noted a significant level of taxonomic novelty within this environment and highlighted the presence of microbial taxa associated with low phosphorus (P) soils, which have the potential for phosphorus turnover [47]. Our in-silico approach involved reducing the microbial community based on the metabolic complementarity between bacterial species and host crop plants, aiming to identify hub species that preserve the essential PGPTs for the design of a SymCom.

We showed that the selection of this minimal community was based on our target compound, encompassing essential compounds for their metabolism, such as amino acids, nucleotide components, cofactors, vitamins, phytohormones, organic acids, and other compounds relevant to plant interactions. Through this process, we successfully reduced the initial community size from 270 to 68 species, resulting in a notable change of approximately 4.5-fold. This reduction is particularly intriguing, considering the intricate microbial interactions typically observed within soil communities [56–58]. Remarkably, the microbial community exhibited significant production of amino acids, organic acids, vitamins, aromatic compounds, and various inorganic ions. These findings align with previous studies on plant microbiomes, which also preferentially utilize these nutrients [19]. These compounds have been extensively documented to play a crucial role in plant–microbe interactions, particularly through root secretions where plants can signal and attract beneficial microbes under specific conditions [59–63]. In contrast, gut microbes were found to predominantly metabolize lipids, sugar derivatives, and carboxy acids [38].

The minimal community we identified preserved important genes associated with PGPTs, including those involved in iron acquisition, EPS production, potassium solubilization, nitrogen fixation, GABA production, and IAA-related tryptophan metabolism. This emphasizes the significance of screening novel microbial taxa, particularly under harsh conditions. The findings suggest that such microbial taxa may assist plants in thriving during drought conditions. Recent studies in the Atacama Desert have highlighted the role of plant growth-promoting bacteria (PGPB) and positive gene selection in facilitating key mechanisms for plant survival [64]. In line with this study, an emphasis on the presence of Acidobacteriota, Eremiobacterota, and Verrucomicrobiota as members of this group of PGPB. It is worth noting that members of these phyla are often characterized as slow-growing bacteria and most lack culture representative. A genome-centric approach has recently provided a comprehensive analysis of 756 MAGs belonging to Acidobacteriota, revealing their potential to promote plant growth through their interactions (Gonçalves et al., [65]).

Within the minimal community, we carefully selected eight hub-species or essential symbiotics that were present in all minimal communities and enabled the production of target metabolites. These hub species were chosen to compose the SymCom and represented four phyla, namely Cyanobacteria, Eremiobacterota, Proteobacteria, and Verrucomicrobiota. In our initial round of species interactions, we excluded two proteobacteria that displayed high competitiveness within SymCom. It has been documented that genome-encoded metabolic potential tends to cluster quantitatively and qualitatively based on phylogeny, resulting in competitive behaviors among species [19, 66]. The exclusion of these species revealed that all the species within the SymCom exhibited cooperative interactions, with a member of the Eremiobacterota phylum playing a supportive role in the metabolism of most species. This finding further underscores the significance of this phylum in microbial interactions. To comprehend the interaction between SymCom and the host, we integrated five important crop plants, including soybean, maize, sorghum, and sugarcane, into our study. Interestingly, our results demonstrated that, in general, only sorghum selected unique microbial species. However, a core set of species remained consistent across different hosts, suggesting their importance independent of the specific plant type. This result was crucial in identifying six hub species that could enhance the growth of all crops. Furthermore, we found that the hosts primarily provided amino acids, lipids, and coenzymes, while the SymCom, in addition to PGPTs, supplied carbohydrates, esters, amino acids, and aromatic compounds to the hosts.

## Conclusion

The soil microbiome associated with plants in stressful environments offers an exceptional opportunity to investigate how plants select beneficial microbial taxa to enhance their survival. It is well-known that soil microbe interactions are complex and depend on various biotic and abiotic factors. Recent advancements in sequencing technologies have allowed for insights into these microbes and their interactions within their natural environment. Computational modeling and prediction approaches enable the exploration of such dynamics.

In this study, we employed an in-silico approach using genome metabolic modeling to design a synthetic microbial community aimed at improving the yield of important crop plants. This approach relied on comprehensive knowledge of microbe-microbe-plant interactions and involved the selection of key species carrying essential plant growth-promoting traits. Similar approaches, as demonstrated here, can be combined with culturomics- and metagenomics-based techniques [67] to design synthetic microbial communities as microbial inoculants for future agricultural production.

### Supplementary Information

**Additional file 1**. Supplementary Figures.

**Additional file 2**.  Supplementary Tables.

## Data Availability

The genomes used in this study are publicly available in the supplementary information. Source data with genes associated with plant growth-promoting traits (PGPTs) in the MAGs and GSMNs file are available in the Zenodo repository: https://doi.org/10.5281/zenodo.8289904. The datasets generated and analyzed during the study are provided in this paper in supplementary information.
