# Supplementary material for: Designing a synthetic microbial community through genome metabolic modeling to enhance plant–microbe interaction
Source: Environ Microbiome. 2023 Nov 16;18:81. doi: 10.1186/s40793-023-00536-3 (PMC10655421; doi:10.1186/s40793-023-00536-3)
Supplement: Supplementary file 1 — Additional file 1. Supplementary Figures. [file 40793_2023_536_MOESM1_ESM.docx]

**Designing a Synthetic Microbial Community through Genome Metabolic Modeling to enhance Plant-Microbe Interaction**

Osiel S. Gonçalves1, Christopher J. Creevey^2^, Mateus F. Santana1*

1Grupo de Genômica Eco-evolutiva Microbiana, Laboratório de Genética Molecular de Microrganismos, Departamento de Microbiologia, Instituto de Biotecnologia Aplicada à Agropecuária, Universidade Federal de Viçosa, Minas Gerais, Brazil.

^2^School of Biological Sciences, Institute for Global Food Security, Queen’s University Belfast, Belfast, BT9 5DL, UK.

## ^*^Correspondence authors: [mateus.santana@ufv.br](mailto:mateus.santana@ufv.br)

**Supplementary Figures**


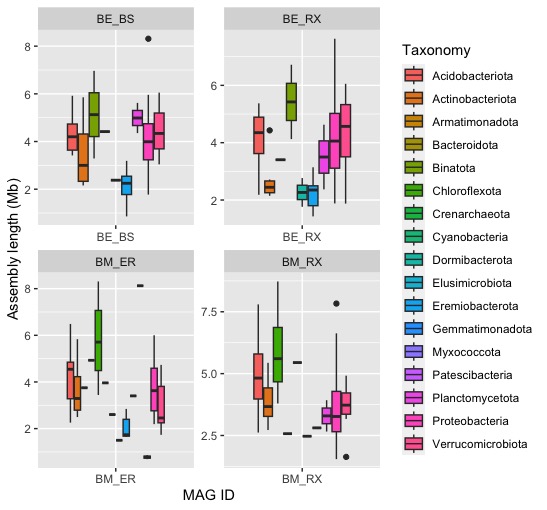

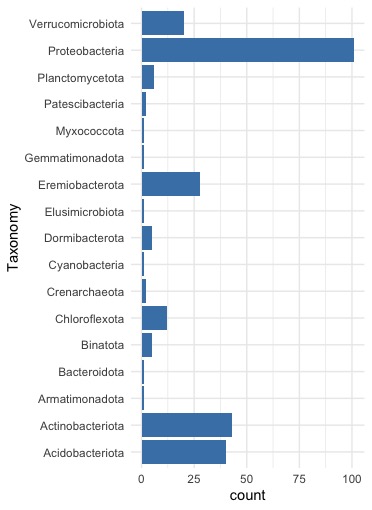


A

B

**Figure S1**. Taxonomic distribution of the soil and rhizosphere microbiome associated with dominant plant species in the Campos rupestres. A. Number of MAGs associated with different Phyla. B. Distribution of MAGs sizes, categorized by Phyla, and their respective patterns observed across the samples. Abbreviations: BE_BS (bulk soil of *V. epidendroides*), BE_RX (Rhizosphere of *V. epidendroides*), BM_BS (bulk soil of *B. macrantha*), and BM_RX (rhizosphere of *B. macrantha*).


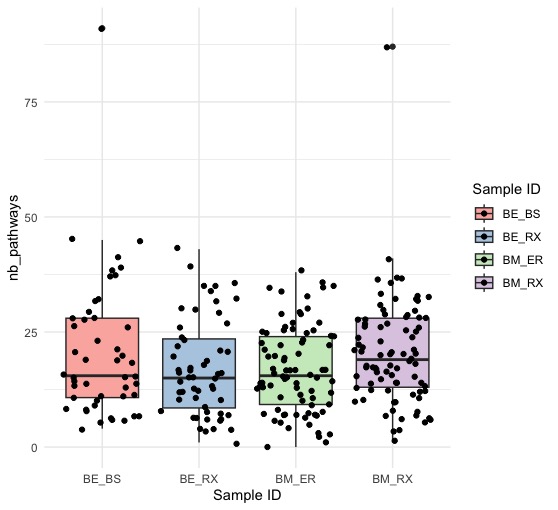


**Figure S2**. Number of pathways reconstructed from 270 MAGs across the samples. Abbreviations: BE_BS (bulk soil of *V. epidendroides*), BE_RX (Rhizosphere of *V. epidendroides*), BM_BS (bulk soil of *B. macrantha*), and BM_RX (rhizosphere of *B. macrantha*).


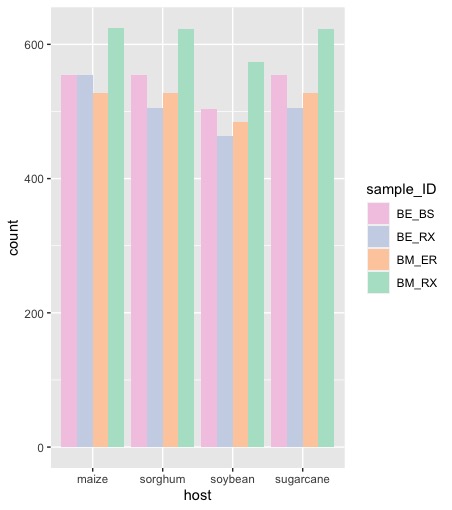


**Figure S3**. Computes scopes for individual metabolic networks across the hosts in different samples. Abbreviations: BE_BS (bulk soil of *V. epidendroides*), BE_RX (Rhizosphere of *V. epidendroides*), BM_BS (bulk soil of *B. macrantha*), and BM_RX (rhizosphere of *B. macrantha*).


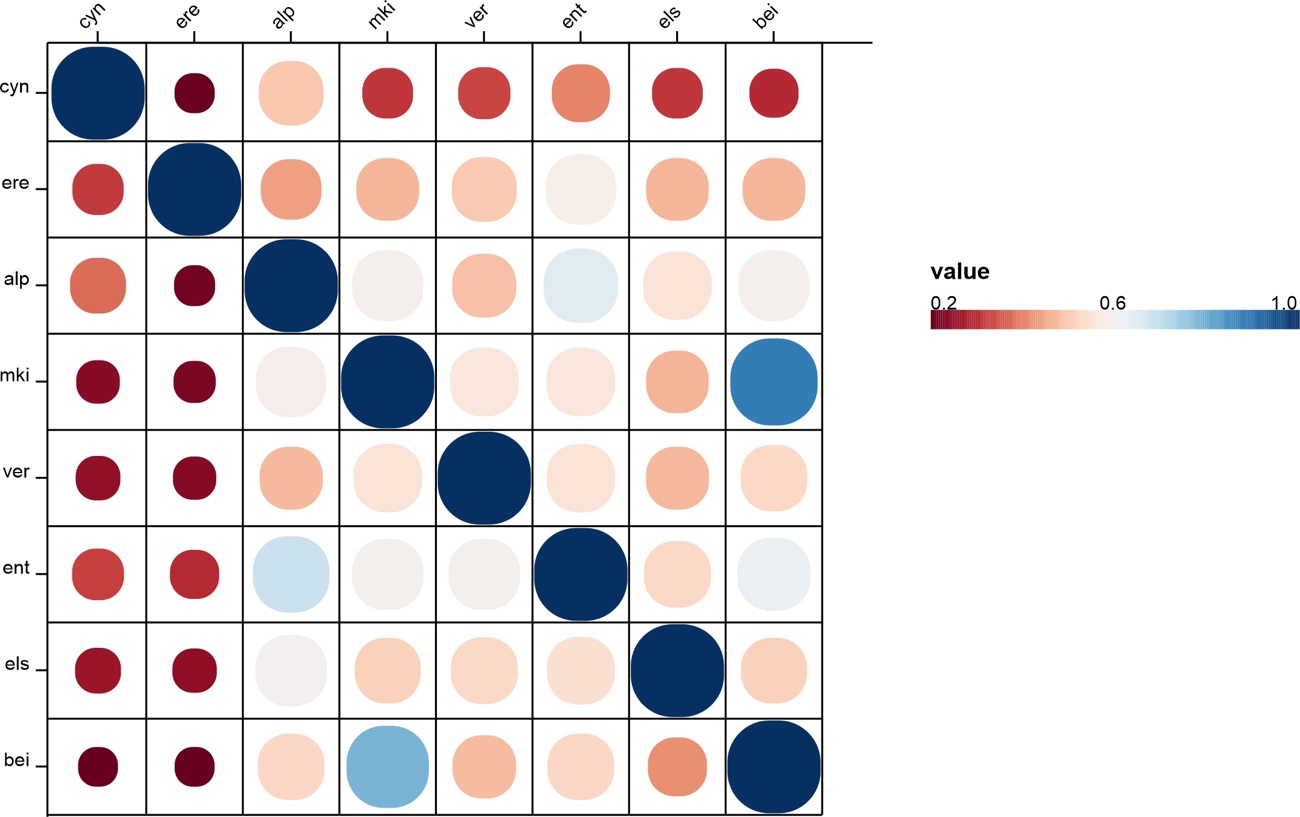


**Figure S4**. Matrix plot of the competition index among six species of SymCom. The competition index ranges from 0 (indicating the lowest competition) to 1 (representing the highest competition index). The size and color of each circle correspond to the competition and complementary index. Abbreviations: alp (Alphaproteobacteria), cya (Cyanobacteria), bei (*Beijerinckiaceae*), els (Elsterales), ent (Enterobacter), ere (Eremiobacterota), mki (*Metakosakonia intestini*), ver (Verrucomicrobiota).


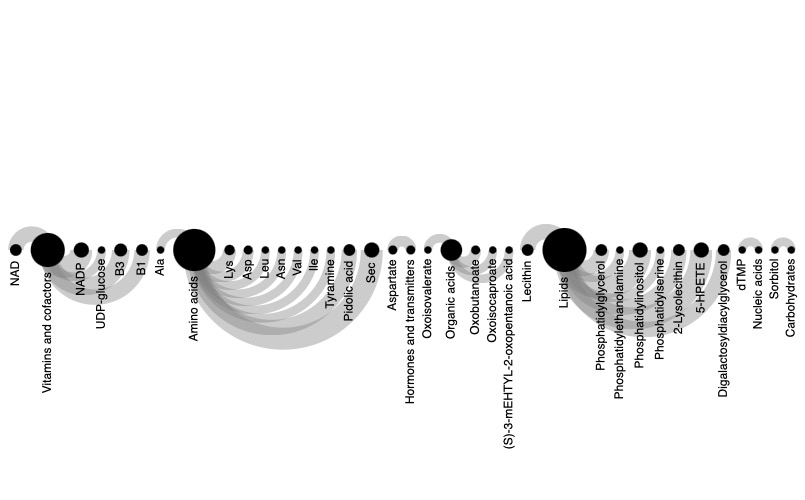


**Figure S5**. Detailed information regarding the compounds in the interaction between hosts and the SymCom. Circles represent the proportion of each class, while arcs depict the set of compounds within each class.
